# Locating Nesting Sites for Critically Endangered Galápagos Pink Land Iguanas (*Conolophus marthae*)

**DOI:** 10.3390/ani14121835

**Published:** 2024-06-20

**Authors:** Marco Gargano, Giuliano Colosimo, Lorenzo Garizio, Paolo Gratton, Gregory A. Lewbart, Glenn P. Gerber, Pierpaolo Loreti, Alexandro Catini, Lorenzo Bracciale, Massimiliano De Luca, Francesca Mastrangeli, Christian Sevilla, Gabriele Gentile

**Affiliations:** 1 PhD Program in Evolutionary Biology and Ecology, Department of Biology, University of Rome Tor Vergata, 00133 Rome, Italy; marco.gargano@alumni.uniroma2.eu (M.G.); lorenzo.garizio@students.uniroma2.eu (L.G.); 2 Department of Biology, University of Rome Tor Vergata, 00133 Rome, Italy; paolo.gratton@uniroma2.it; 3 College of Veterinary Medicine, North Carolina State University, 1060 William Moore Drive, Raleigh, NC 27606, USA; galewbar@ncsu.edu; 4 UNC-Chapel Hill Galapagos Science Center (GSC), Universidad San Francisco de Quito (USFQ), Av. Alsacio Northia, Quito 170901, Ecuador; 5 San Diego Zoo Wildlife Alliance, 15600 San Pasqual Valley Road, Escondido, CA 92027, USA; ggerber@sdzwa.org; 6 Department of Engineering, University of Rome Tor Vergata, 00133 Rome, Italy; pierpaolo.loreti@uniroma2.it (P.L.); catini@ing.uniroma2.it (A.C.); lorenzo.bracciale@uniroma2.it (L.B.); 7 Italian National Council of Research, Institute of Marine Engineering, 00133 Rome, Italy; massimiliano.deluca@cnr.it; 8 Italian National Council of Research, Institute for Microelectronics and Microsystems, 00133 Rome, Italy; francesca.mastrangeli@cnr.it; 9 Galápagos National Park Directorate, Av. Charles Darwin—200102 Puerto Ayora, Is. Santa Cruz, Galápagos 200102, Ecuador; csevilla@galapagos.gob.ec

**Keywords:** net displacement (ND), generalized additive mixed model (GAMM), head-start program, movement pattern, nesting behavior, hatchlings, conservation

## Abstract

**Simple Summary:**

Galápagos pink land iguanas (*Conolophus marthae*) are a critically endangered species endemic to Wolf Volcano, Galápagos. Among other conservation initiatives, a head-start program, involving the captive rearing of hatchlings prior to release in the wild, has been identified as a primary action needed to prevent the species’ extinction. However, until this study began, the location of pink iguana nesting grounds was unknown, and no hatchlings and only a handful of juveniles and subadults had been observed. In an attempt to unveil the location of nesting grounds for *C. marthae*, we modeled the movement patterns of male and female iguanas tracked during the reproductive season. Based on the hypothesis that males and females might exhibit different movement patterns after the breeding season, we looked for females-specific migratory behavior. Thanks to this approach, we identified an area that females likely use to nest. Our results based on movement data alone led to the first-ever identification of pink iguana nests and hatchlings.

**Abstract:**

Invasive alien species control is recognized worldwide as a priority action to preserve global biodiversity. However, a lack of general life history knowledge for threatened species can impede the effectiveness of conservation actions. Galápagos pink land iguanas (*Conolophus marthae*) are endemic to Wolf Volcano, Galápagos, Ecuador. These iguanas are threatened by invasive alien species, particularly feral cats, that may affect their small population size. To guarantee the long-term survival of *C. marthae*, the Galápagos National Park Directorate is considering, along with an ongoing campaign of feral cat control, the implementation of a head-start program. However, the success of this management strategy necessarily relies on the identification of pink iguana nesting grounds, which were still unknown at the onset of this study. We modeled the movement patterns of male and female iguanas during the reproductive season, using location data collected from custom-made remote tracking devices installed on adult pink iguanas in April 2021. We first calculated for each individual the vector of distances from its starting location, which was defined as net displacement. We then used net displacement as the response variable in a generalized additive mixed model with day of the year as the predictor. Based on the hypothesis that males and females may behaviorally differ after mating, we looked for female-specific migratory behavior suggesting females were moving toward nesting areas. The results obtained confirmed our hypothesis, as females exhibited a distinct migratory behavior, reaching a small plateau area inside of Wolf Volcano’s caldera and ca. 400 m below the volcano’s northern rim. Moreover, once inside the caldera, females displayed a more aggregated distribution pattern. The movement data obtained allowed Galápagos National Park rangers to locate individual pink iguana nests and subsequently to sight and collect the first observed hatchlings of the species. This work constitutes a necessary baseline to perform dedicated studies of pink iguana nests and emerging hatchling iguanas, which is an essential step toward the development of an effective head-start program.

## 1. Introduction

Island ecosystems, for the area they occupy, host a disproportionately large amount of global biodiversity compared to continental ecosystems [1]. Tropical islands, in particular, are among the most critical biodiversity hotspots of the world due to their unique climatic and environmental conditions and huge concentration of endemic species [2,3,4]. The biodiversity of these fragile ecosystems is highly vulnerable to human-driven pressures including climate change, habitat degradation and the introduction of invasive alien species (IASs) [4,5,6]. Overall, IASs are estimated to be one of the main drivers of vertebrate extinctions in island ecosystems, and IASs control has been identified as one of the main targets for safeguarding global biodiversity [6]. However, the lack of biological knowledge about endemic species may hamper the development of successful conservation actions [7]. A deeper knowledge of native species ecology is necessary to understand how IASs may affect them and to inform management authorities.

The Galápagos archipelago represents one of the world’s most iconic island ecosystems and harbors many unique animal species [8]. Galápagos Pink Land Iguanas (*Conolophus marthae*) are one of three terrestrial iguana species endemic to the archipelago [9,10,11]. Only described in 2009 [12], *C. marthae* were rapidly assessed as being Critically Endangered by the IUCN Red List of Threatened Species [9]. Only a single population of about 200 adult pink iguanas exists [13], which was confined to the northwestern slope of Wolf Volcano on Isabela Island [14]. Data collected in the field over sixteen years provided fundamental insights on the ecology of this species [13,15,16]. However, hatchlings were never encountered, and juveniles were rarely observed [14]. Therefore, quantitative and ecological information on pink iguana early-life stages are dramatically scarce. There are no native predators of terrestrial iguanas on Wolf Volcano except for Galápagos Hawks (*Buteo galapagoensis*). However, invasive black rats and feral cats occur at this site [9,14]. They are potential predators of early-stage individuals [9,17,18]. Recent analyses demonstrated that population recruitment, although extremely limited, has been sufficient to prevent measurable population decline [13]. Nonetheless, it is likely that IASs affect population dynamics on a long-term scale, conditioning the limited recruitment observed. Because of this, editors of the 2022–2027 Conservation and Management Plan (CAMP) for the species [19] identified IASs control as one of the CAMP’s high-priority actions. Concurrently, the Galápagos National Park Directorate (GNPD) is evaluating the feasibility of a head-start program for the species [20]. However, the current lack of information about nesting and hatchling ecology hampers the realization of these conservation actions. Even more concerning, until this study began, the location of pink iguana nesting areas was unknown, which was mainly due to the remoteness of the site, making it logistically difficult and expensive to conduct field studies of significant duration.

Given the above-mentioned logistic issues, our research group designed and developed a custom-made GPS Wireless Sensor Node (WSN) to remotely track iguanas in the wild [21,22]. We installed the first tracking devices on pink iguanas in September 2019, providing preliminary information about the movement ecology of the species [15].

In the present study, we analyzed the location data collected by WSNs installed on iguanas in April 2021, when females begin to be reproductively active [23], in the attempt to identify nesting areas for the species. We described the path followed by 11 pink iguana females tagged during the mating season, and we used two tagged males as control. We assessed whether the variation in individual movements observed may be explained in terms of differences in sex-specific movement patterns. Female iguanas of many species are known to migrate from their usual home ranges to reach open areas to dig nests [24,25,26,27,28,29]. Hence, we anticipated that female pink iguanas might display migratory behavior during the study period while looking for suitable areas to nest. On the contrary, males should remain closer to their usual activity centers, as expected for individuals moving within a stable home range [30].

We are aware that the reduced sample size of this study does not allow extrapolating our findings to the entire population. However, the analysis proposed here represents the first attempt to identify the location of nesting grounds for the species and could provide preliminary information about the timing, direction, and extent of the nesting migration.

## 2. Materials and Methods

### 2.1. Data Collection and Sampling Area

**Figure 1:**
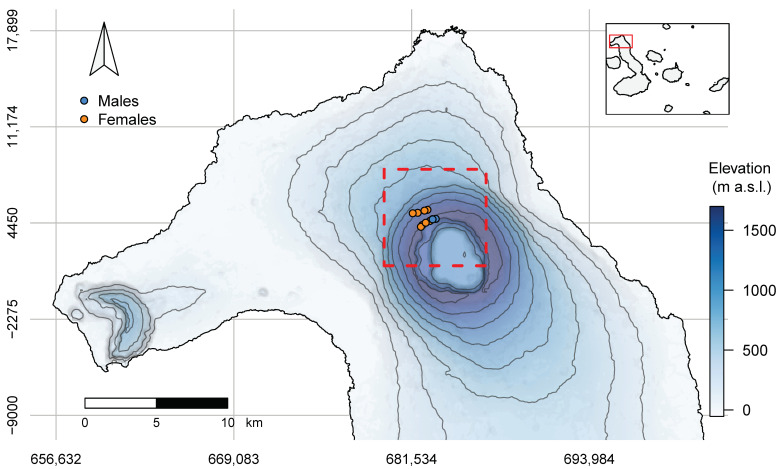
Topographic map of the study area, located on the northwestern slope of Wolf Volcano, Isabela Island. The main map shows the location of the study area (dashed red line), whose relative position within the Galápagos archipelago is shown in the inset (red square). Capture points of male (blue) and female (orange) pink iguanas equipped with Wireless Sensor Nodes (WSNs) are shown in the main map. The continuous blue palette used in the main map represents the elevation (meters above sea level) with darker shades of blue corresponding to higher altitudes. Gray contour lines represent 200 m elevation changes.

In April 2021, we captured 13 female and 2 male Galápagos Pink Land Iguanas. We equipped each iguana with a WSN [15,21,22]. All iguanas were captured between 2 April and 5 April on the outer slopes of Wolf Volcano near the rim, within an area not larger than 2 km^2^, ranging between 1390 and 1615 m above sea level (a.s.l.) (Figure 1).

Mating season occurs approximately between April and June, when pink iguanas congregate at the top of Wolf Volcano to breed [14]. The mating area is mainly covered by highland deciduous grass communities interspersed with patches of seasonal shrubland [31]. All sampled individuals were checked for the presence of Passive Integrated Transponders (PITs) applied during past field trips, and individuals captured for the first time were tagged with a new unique PIT. For each iguana, we recorded the coordinates of the capture point, sex, and morphometric features of interest (e.g., snout-to-vent length, SVL). We also assessed the reproductive status of females using a SonoSite portable ultrasound machine (FUJIFILM SonoSite, Inc., Bothell, WA, USA) to determine the presence and number of developing eggs (Table 1).

WSNs were attached with a combination of epoxy glue and absorbable synthetic stitches. A board-certified veterinarian performed suturing. All capture and handling procedures were approved by the Galápagos National Park Directorate (research permit #PC-04-21) and carried out in the presence of park rangers.

### 2.2. Data Filtering

Before the analysis, we assessed the accuracy of GPS points based on the Horizontal Dilution of Precision (HDOP) factor [32]. This is a metric associated with the relative position of satellites to the receiver. It can be used to measure the accuracy of GPS fixes. As lower values of HDOP indicate higher precision, we kept only GPS fixes with an HDOP ≤ 1.4 as specified in [22]. We additionally filtered the data by date to retain only those locations collected during the period of interest, April through June. We then removed individuals that had less than 20 recorded GPS fixes. Our final dataset consisted of 1539 data points belonging to 13 WSNs (11 attached to females and two to males). All females that passed the filtering procedure had eggs at various developmental stages (Table 1).

### 2.3. Statistical Analysis

Different path metrics can be used to infer animal behavior from movement trajectories. For example, step length, turning angles, and spatial displacement are some of the primary derived parameters that are widely used in movement ecology [33]. Many studies have adopted these types of measures as proxies of animal movement, proving their reliability in differentiating between alternative behavioral states, e.g., migration vs. sedentarism [34,35,36,37,38]. In this study, we summarized the movement path of each individual computing the vectors of net displacement (ND) values. We calculated ND values for each individual as the vector of linear distances in meters between the first location and each subsequent relocation. We assessed differences in the movement of males and females by fitting one generalized additive mixed model (GAMM) with Tweedie error distribution and log link function. We set ND as the response variable and a thin plate regression spline of the day of the year as the predictor. We modeled the smooth effect of time (the day of the year) for each sex separately. As each separate smooth effect is centered around zero, sex was also included as a parametric term to account for differences in the mean values of ND between males and females. Smoothness selection was performed via restricted maximum likelihood (REML). To account for the non-independence of data points recorded by the same device, we also included the effect of time for each individual as a non-linear random effect (hereafter referred to as random smooth). The significance of adding sex to the final model was tested using a likelihood ratio test (LRT) between the full model and a nested model including only a general thin plate spline of time and the random smooths. All statistical analyses were performed in R version 4.2.2 [39]. GAMMs were fitted using R package *mgcv* [40], and likelihood ratio tests were performed using the *lrtest()* function of the *lmtest* R package [41].

## 3. Results

Table 2 shows the results of the GAMM fitted to estimate the combined effect of time and sex on ND values calculated for each GPS fix.

**Figure 2:**
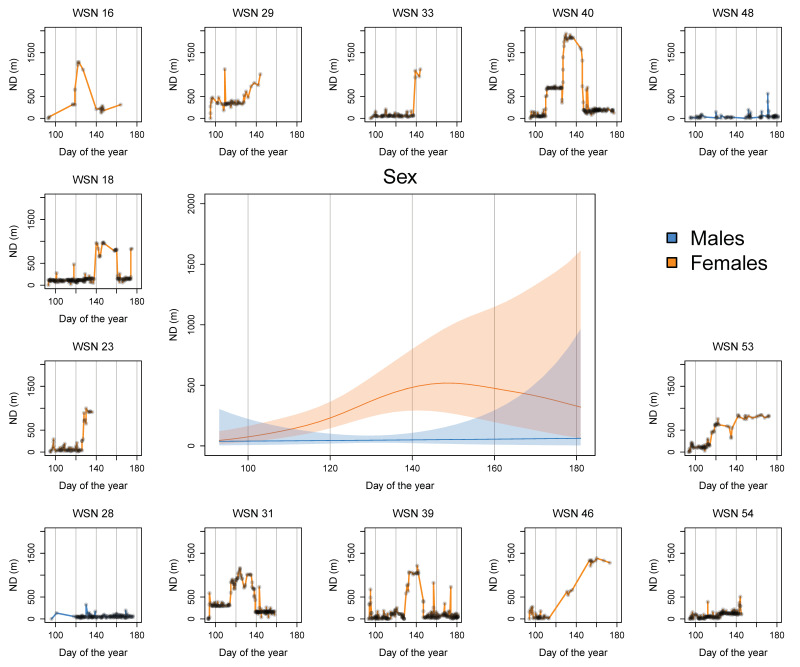
Generalized additive mixed model (GAMM) fitted smoothing splines and their estimated 95% confidence intervals, describing trends of net displacement (ND) values over time. The central panel shows fitted trends of ND over time for males (blue) and females (orange). Analogous plots around the edges of the main figure show the ND values calculated for the location data collected by each tracking device included in the analysis.

We found a statistically significant difference in ND values calculated for males and females over time. During the entire study period, ND values for males were always close to zero (Figure 2; Table S1), as predicted for animals patrolling a restricted area [30].

On the contrary, ND smoothing splines of females over time strongly differed from a flat horizontal line (Figure 2). While ND values were closer to zero at the beginning of the study period, they strongly increased by the middle of the study period as expected for migrating animals [30]. Sex-specific splines significantly differed between the end of April and the beginning of June when the peak of migration took place (Figure 2).

Four females dispersed less than 1 km from their starting location, while all other females dispersed longer distances (Table S1). One female moved about 2 km away from her starting location, which was the maximum migration observed. At the end of the study period, the mean ND values for females decreased again, although the confidence interval was much larger due to the lack of data from specific devices (Figure 2).

## 4. Discussion

In the present study, we analyzed seasonal location data collected with custom-made tracking devices developed to monitor critically endangered Galápagos pink land iguanas. We fitted two male and 13 female pink iguanas with tracking devices at the beginning of the mating season. Our primary goal was to identify the potential location of nesting areas for the species. This approach led us to statistically identify movement patterns that differentially characterized males and females of the species during the reproductive season.

**Figure 3:**
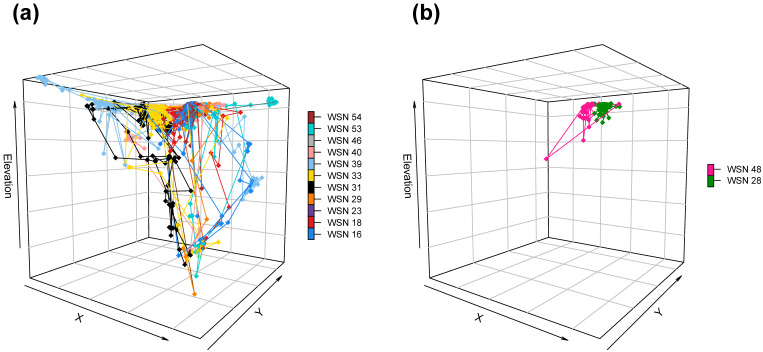
Movement trajectory of female (**a**) and male (**b**) iguanas tracked during the sampling period. Each line connects the location data points recorded by a single iguana. Explicit values of GPS UTM coordinates (X and Y) and Elevation were omitted from the graph to avoid disclosing the exact locations of female nesting sites, but the geographical space represented in the 3D plots is exactly the same.

**Figure 4:**
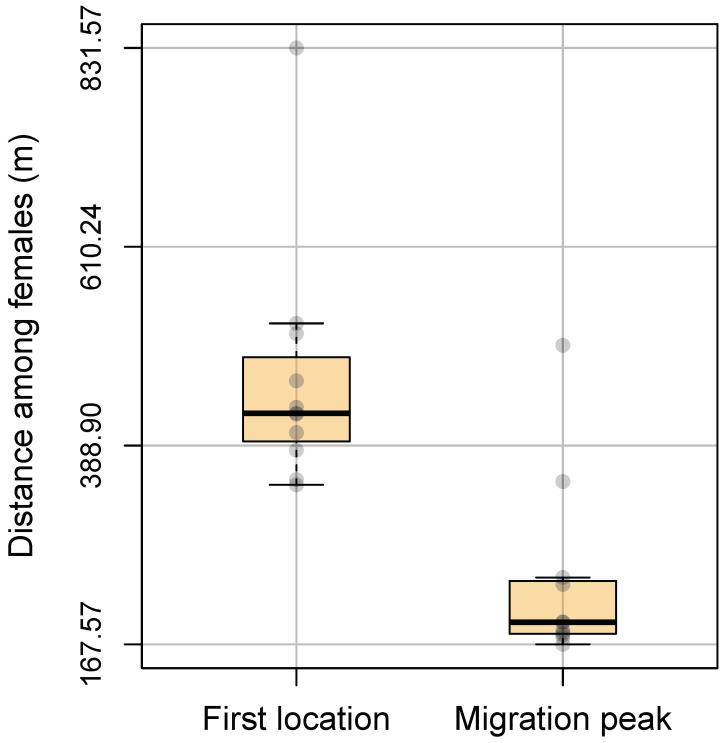
Boxplots showing the average pairwise distance between females at the beginning of the sampling period and during the migration peak. This figure shows how females aggregated during the migration peak. The boxplot on the left shows the average pairwise distance between females at their first location. The boxplot on the right shows the same variable calculated at the peak of migration for each female. For each female, migration peak has been defined as the lowest elevation position occupied during the study period. Semi-transparent dots represent the average pairwise distance calculated for each female.

At the beginning of the sampling period, males and females remained close to their original capture location, and their movements were not discernible. At the end of April, females started to migrate far from their respective capture locations. Although migration started at a different time for each female (Figure 2), we observed a migratory peak in May (Table S1). During this migration, females travelled up to ca. 2 km to reach a single common area (Figure 3 and Figure 4; Table S1) not larger than 2 km^2^ where they spent anywhere from a few days to one or two weeks before returning to the vicinity of their respective original capture locations (Figure 2 and Figure 3).

While tagged females were migrating to the presumed nesting area, tagged males did not change their movement patterns. Indeed, the two tagged males spent almost the entire reproductive season within small areas on the rim of Wolf Volcano, as ND values recorded for these iguanas remained consistently close to zero (Figure 2; Table S1).

The short-term migration described for females is consistent with expectations of nesting behavior. Female iguanas of several species are known to migrate kilometers to reach suitable areas to dig nests [24,25,26,27,28,29]. Immediately after laying eggs, female iguanas of many species spend up to three weeks guarding their nests, probably to defend the area from other nesting females [28,29,42]. Therefore, the time *C. marthae* females spent at their destination (Figure 2) may be interpreted as a combination of nest site selection, nesting (excavation, oviposition, and closure), and perhaps nest guarding to reduce nest failure due to intraspecific competition. However, it is important to emphasize that our results suggest that female pink iguanas did not appear to spend much time guarding their nests, which was similar to observations of nesting *Iguana delicatissima* females in Dominica [43].

The above-mentioned similarities with the nesting behavior of other large iguanas offered us clear insights into the potential location of a nesting area for pink iguanas, corresponding to the common destination of paths taken by tracked pink iguana females (Figure 3 and Figure 4). Geographically, this corresponds to a small plateau located inside the north side of Wolf Volcano caldera and ca. 400 m below the volcano’s rim. The localization of a nesting area inside the caldera of a volcano is not uncommon among *Conolophus* iguanas [11,25]. On the nearby island of Fernandina, female *C. subcristatus* have been observed digging nests inside the caldera of La Cumbre volcano, probably to take advantage of geothermal heat near fumaroles [25]. Females may select nesting sites with warmer temperatures to shorten the incubation time of eggs and reduce the predation risk to nests [25,44,45,46]. Scientists observed similar behavior on Wolf Volcano, where *C. subcristatus* females excavate nests on the floor of the caldera [11]. The potential nesting area that we identified is also characterized by low vegetation cover. Once again, this aspect is not uncommon for large iguanas, as these reptiles usually construct nests in low-vegetated open areas with soft soil, where it is easier to dig and nests receive maximum solar radiation [26,28,43,47].

Although the reduced sample size of our study demands caution in extending the described behavioral patterns to the entire population, this is the first attempt to quantitatively describe *C. marthae* sex-specific behaviors during the reproductive season. Even more importantly, the results presented here provided evidence for the identification of a potential nesting area for this species. Our data were relayed to the Galápagos National Park Directorate, which implemented specific actions to monitor the identified area. This strategy led to the first-ever identification of *C. marthae* nests and to the first direct observation of pink iguana hatchlings (https://galapagos.gob.ec/neonatos-de-iguana-rosada-fueron-descubiertos-por-primera-vez-en-isla-isabela/ (accessed on 15 June 2024)).

The results obtained were, therefore, fundamental in assisting management actions and are pivotal for developing future conservation strategies. Galápagos iguanas have evolved on isolated islands where they experienced minimal predation pressures and are therefore more prone to predation by invasive alien mammals [48]. Although feral cats do not pose a significant threat to the adults of most large iguana species, they actively prey on hatchlings and juveniles [9,17,18]. For these reasons, a project for the control of these introduced mammals on Wolf Volcano is now ongoing [19]. In this context, the results obtained may provide useful information to optimize actual control protocols, prioritizing efforts focused on the nesting area identified inside the Wolf Volcano caldera. Accordingly, a parallel plan to actively locate, protect and monitor nests should be implemented from the end of the laying season until hatchlings emerge and disperse to reduce predation by introduced mammals and obtain more information on *C. marthae* reproductive ecology. Our findings call for a deeper investigation into the drivers of nest site selection and fidelity. Additionally, they point out the importance of investigating the different phases of *C. marthae* reproduction from egg deposition to hatchling dispersal. Collecting information about egg incubation conditions and hatching success along with hatchling emergence dates and dispersal behavior is indeed crucial to inform management authorities and to evaluate the feasibility of specific conservation actions such as a head-start or captive breeding program being considered by the GNPD.

## 5. Conclusions

Although detailed information about the nesting ecology and hatchling requirements of *C. marthae* are still pending, this work constitutes the first attempt to quantitatively estimate the movement patterns of males and females during the reproductive season. With the localization of a species nesting ground, this works provides crucial information to focus specific management strategies, including invasive predator control and the monitoring of individual nest sites, within the identified species nesting area. In addition, our results provide essential information needed to perform dedicated studies of newly emerged hatchlings and to evaluate the development of an effective head-start program.

## Figures and Tables

**Table 1:** Table of the WSNs attached during the April 2021 field trip. Individuals marked with an asterisk were not retained for the analysis. The table presents for each WSN: the attachment date (Date), sex of the tagged individual (Sex: M for male, F for female), number of developing eggs recorded for females (Eggs), and the number of points transmitted that passed the filtering procedure (Points, see Section 2.2).

WSN	Date	Sex	Eggs	Points
WSN 16	2021-04-04	F	2	23
WSN 18	2021-04-02	F	2	199
WSN 23	2021-04-02	F	2	70
WSN 28	2021-04-02	M	-	137
WSN 29	2021-04-03	F	5	51
WSN 31	2021-04-05	F	2	170
WSN 33	2021-04-04	F	2	82
WSN 39	2021-04-03	F	4	231
WSN 40	2021-04-04	F	2	189
WSN 46	2021-04-02	F	2	49
WSN 47 *	2021-04-03	F	0	5
WSN 48	2021-04-02	M	-	89
WSN 53	2021-04-02	F	2	86
WSN 54	2021-04-03	F	2	163
WSN 58 *	2021-04-02	F	2	11

**Table 2:** Model output from the generalized additive mixed model (GAMM) describing the pattern of net displacement (ND) over time. The table shows the results of the GAMM fitted to assess differences in ND between male and female pink iguanas during the reproductive season. The table shows the estimated coefficient (Estimate) for each model term with the corresponding standard error (S.E.), degrees of freedom (*df*) or effective degrees of freedom (*edf*, for smooth terms), and the F statistic (F) with corresponding probability values (*p*-values). F statistics and corresponding *p* values for intercepts are uninterpretable.

**Parametric Coefficients**					
**Term**	Estimate	S.E.	*F*	*df*	*p*-value
**Intercept**	5.4795	0.1398	-	-	
**sex: M**	−1.6294	0.3321	24.07	1	0.001
**Smooth terms**					
**Term**			*F*	*edf*	*p*-value
**s (time): sex F**			3.669	3.227	0.008
**s (time): sex M**			0.044	1.001	0.834
**s (time, WSN)**			35.105	84.759	0.001

## Data Availability

Dataset available upon request to the authors.

## Supplementary Materials

The following supporting information can be downloaded at https://www.mdpi.com/article/10.3390/ani14121835/s1, Table S1: Summary table showing principal morphometrics and path metrics for each tagged iguana.

## References

[1] Fernández-Palacios J.M., Kreft H., Irl S.D.H., Norder S., Ah-Peng C., Borges P.A.V., Burns K.C., de Nascimento L., Meyer J.Y., Montes E. (2021). Scientists’ warning—The outstanding biodiversity of islands is in peril. Glob. Ecol. Conserv..

[2] Myers N., Mittermeier R.A., Mittermeier C.G., da Fonseca G.A.B., Kent J. (2000). Biodiversity hotspots for conservation priorities. Nature.

[3] Perfecto I., Vandermeer J. (2008). Biodiversity conservation in tropical agroecosystems: A new conservation paradigm. Ann. N. Y. Acad. Sci..

[4] Gillespie T.W., O’Neill K., Keppel G., Pau S., Meyer J.Y., Price J.P., Jaffré T. (2014). Prioritizing conservation of tropical dry forests in the Pacific. Oryx.

[5] Fordham D.A., Brook B.W. (2010). Why tropical island endemics are acutely susceptible to global change. Biodivers. Conserv..

[6] Spatz D.R., Zilliacus K.M., Holmes N.D., Butchart S.H.M., Genovesi P., Ceballos G., Tershy B.R., Croll D.A. (2017). Globally threatened vertebrates on islands with invasive species. Sci. Adv..

[7] Sutherland W.J., Pullin A.S., Dolman P.M., Knight T.M. (2004). The need for evidence-based conservation. Trends Ecol. Evol..

[8] Steinfartz S., Zachos F.E., Habel J.C. (2011). When hotspots meet: The Galápagos islands: A hotspot of species endemism based on a volcanic hotspot centre. Biodiversity Hotspots: Distribution and Protection of Conservation Priority Areas.

[9] Gentile G. (2012). *Conolophus* *marthae*. The IUCN Red List of Threatened Species 2012: e.T174472A1414375.

[10] Gentile G., Grant T.D. (2020). *Conolophus* *pallidus*. The IUCN Red List of Threatened Species 2020: e.T5239A3014028.

[11] Kumar K., Gentile G., Grant T.D. (2020). *Conolophus* *subcristatus*. The IUCN Red List of Threatened Species 2020: e.T5240A3014082.

[12] Gentile G., Snell H. (2009). *Conolophus marthae* sp.nov. (Squamata, Iguanidae), a new species of land iguana from the Galápagos archipelago. Zootaxa.

[13] Garizio L., Gargano M., Colosimo G., Gratton P., Gerber G.P., Lewbart G.A., Sevilla C., Gentile G. (2024). First evidence of recruitment in critically endangered Galapagos pink land iguanas (*Conolophus marthae*). Conserv. Sci. Pract..

[14] Gentile G., Marquez C., Snell H.L., Tapia W., Izurieta A., Angelici F.M. (2016). Conservation of a New Flagship Species: The Galápagos Pink Land Iguana (*Conolophus marthae* Gentile and Snell, 2009). Problematic Wildlife.

[15] Colosimo G., Gargano M., Loreti P., Bracciale L., De Luca M., Catini A., Di Natale C., Vera C., Sevilla C.R., Gerber G.P. (2022). Remote tracking of Galápagos pink land iguana reveals large elevational shifts in habitat use. J. Nat. Conserv..

[16] Gargano M., Colosimo G., Gratton P., Marta S., Brilli M., Giustini F., Sevilla C., Gentile G. (2022). Nitrogen and carbon stable isotope analysis sheds light on trophic competition between two syntopic land iguana species from Galápagos. Sci. Rep..

[17] MacLeod A., Cooke S.C., Trillmich F. (2020). The spatial ecology of invasive feral cats Felis catus on San Cristóbal, Galápagos: First insights from GPS collars. Mammal Res..

[18] Perry G., Knapp C.R., Grant T.D., Pasachnik S.A., Coman I. (2021). From pets to threats: Invasive iguanas and other species cause significant harm to native iguanas. Reptil. Amphib..

[19] Rueda D., Castaño P.A., Campbell K.J., Colosimo G., Gerber G.P., León P., Tapia W., Gentile G. (2023). Galápagos Pink Land Iguana (Conolophus marthae): Conservation and Management Plan 2022–2027.

[20] IUCN/SSC (2013). Guidelines for Reintroductions and Other Conservation Translocations.

[21] Loreti P., Catini A., De Luca M., Bracciale L., Gentile G., Di Natale C. (2019). The Design of an Energy Harvesting Wireless Sensor Node for Tracking Pink Iguanas. Sensors.

[22] Loreti P., Bracciale L., Colosimo G., Vera C., Gerber G.P., De Luca M., Gentile G. (2020). Assessment and validation of miniaturized technology for the remote tracking of critically endangered Galápagos pink land iguana (*Conolophus marthae*). Anim. Biotelemetry.

[23] Onorati M., Sancesario G., Pastore D., Bernardini S., Carrión J.E., Carosi M., Vignoli L., Lauro D., Gentile G. (2016). Plasma concentrations of progesterone and estradiol and the relation to reproduction in Galápagos land iguanas, *Conolophus marthae* and *C. subcristatus* (Squamata, Iguanidae). Anim. Reprod. Sci..

[24] Christian K.A., Tracy C.R., Burghardt G.M., Rand A.S. (1982). Reproductive behavior of Galápagos land iguanas, Conolophus pallidus, on Isla Santa Fe, Galápagos. Iguanas of the World: Their Behavior, Ecology, and Conservation.

[25] Werner D.I., Burghardt G.M., Rand A.S. (1982). Social Organization and Ecology of Land Iguanas, *Conolophus subcristatus*, on Isla Fernandina, Galápagos. Iguanas of the World: Their Behavior, Ecology, and Conservation.

[26] Iverson J.B., Kirsten N.H., Jennifer M.V. (2004). The Nesting Ecology of the Allen Cays Rock Iguana, *Cyclura cychlura inornata* in the Bahamas. Herpetol. Monogr..

[27] Krysko K.L., Enge K.M., Donlan E.M., Seitz J.C., Golden E.A. (2007). Distribution, natural history, and impacts of the introduced green iguana (*Iguana iguana*) in Florida. Iguana.

[28] Perez-Buitrago N., Sabat A.M., McMillan W.O. (2016). Nesting migrations and reproductive biology of the Mona Rhinoceros Iguana, *Cyclura stejnegeri*. Herpetol. Conserv. Biol..

[29] Moss J.B., Gerber G.P., Goetz M., Haakonsson J.E., Harvey J.C., Laaser T., Welch M.E. (2020). Contrasting patterns of movement across life stages in an insular iguana population. J. Herpetol..

[30] Bunnefeld N., Börger L., van Moorter B., Rolandsen C.M., Dettki H., Solberg E.J., Ericsson G. (2011). A model-driven approach to quantify migration patterns: Individual, regional and yearly differences. J. Anim. Ecol..

[31] Rivas-Torres G.F., Benítez F.L., Rueda D., Sevilla C., Mena C.F. (2018). A methodology for mapping native and invasive vegetation coverage in archipelagos. Prog. Phys. Geogr..

[32] Langley R.B. (1999). Dilution of Precision. GPS World.

[33] Edelhoff H., Signer J., Balkenhol N. (2016). Path segmentation for beginners: An overview of current methods for detecting changes in animal movement patterns. Mov. Ecol..

[34] Fryxell J.M., Hazell M., Börger L., Dalziel B.D., Haydon D.T., Morales J.M., McIntosh T., Rosatte R.C. (2008). Multiple movement modes by large herbivores at multiple spatiotemporal scales. Proc. Natl. Acad. Sci. USA.

[35] Gautestad A.O., Loe L.E., Mysterud A. (2013). Inferring spatial memory and spatiotemporal scaling from GPS data: Comparing red deer *Cervus elaphus* movements with simulation models. J. Anim. Ecol..

[36] Singh N.J., Allen A.M., Ericsson G. (2016). Quantifying migration behaviour using net squared displacement approach: Clarifications and caveats. PLoS ONE.

[37] Hertel A.G., Niemelä P.T., Dingemanse N.J., Mueller T. (2020). A guide for studying among-individual behavioral variation from movement data in the wild. Mov. Ecol..

[38] van de Kerk M., Larsen R.T., Olson D.D., Hersey K.R., McMillan B.R. (2021). Variation in movement patterns of mule deer: Have we oversimplified migration?. Mov. Ecol..

[39] R Core Team (2022). R: A Language and Environment for Statistical Computing.

[40] Wood S.N. (2017). Generalized Additive Models: An Introduction with R.

[41] Zeileis A., Hothorn T. (2002). Diagnostic Checking in Regression Relationships. R News.

[42] Wikelski M., Nelson K. (2004). Conservation of Galápagos marine iguanas (*Amblyrhynchus cristatus*). Iguana.

[43] Knapp C.R., Prince L., James A. (2016). Movements and nesting of the Lesser Antillean iguana (*Iguana delicatissima*) from Dominica, West Indies: Implications for conservation. Herpetol. Conserv. Biol..

[44] Shine R., Madsen T.R.L., Elphick M.J., Harlow P.S. (1997). The Influence of Nest Temperatures and Maternal Brooding on Hatchling Phenotypes in Water Pythons. Ecology.

[45] Du W., Ji X. (2006). Effects of constant and fluctuating temperatures on egg survival and hatchling traits in the northern grass lizard (*Takydromus septentrionalis*, Lacertidae). J. Exp. Zool. Part A Comp. Exp. Biol..

[46] Li S., Hao X., Wang Y., Sun B., Bi J., Zhang Y., Janzen F.J., Du W. (2018). Female lizards choose warm, moist nests that improve embryonic survivorship and offspring fitness. Funct. Ecol..

[47] Carreras-De León R., Pasachnik S.A., Gerber G.P., Brooks C.P., Rupp E., Welch M.E. (2019). Genetic structure at three spatial scales is consistent with limited philopatry in Ricord’s Rock Iguanas (*Cyclura ricordii*). Ecol. Evol..

[48] Berger S., Wikelski M., Romero L.M., Kalko E.K., Rödl T. (2007). Behavioral and physiological adjustments to new predators in an endemic island species, the Galápagos marine iguana. Horm. Behav..

