# Supplementary material for: Locating Nesting Sites for Critically Endangered Galápagos Pink Land Iguanas (Conolophus marthae)
Source: Animals (Basel). 2024 Jun 20;14(12):1835. doi: 10.3390/ani14121835 (PMC11200735; doi:10.3390/ani14121835)
Supplement: Supplementary file 1 [file animals-14-01835-s001.zip › animals-3060200-supplementary.pdf]

animals

MDPI

Table S1. Summary table showing principal morphometrics and path metrics for each tagged iguana. The table presents for each WSN: sex (Sex: F for female, M for male), snout-to-vent length (SVL, in cm) and mass (Mass, in kg) of each tagged individual, number of effective tracking days (Days), mean, standard deviation (SD) and maximum value (Max) of Net Displacement (ND, in meters), mean and standard deviation (SD) of elevation (in meters above sea level) occupied by each tracked iguana during the sampling period, and the date of the migration peak for females (Peak date). The migration peak date was defined as the date when each female reached the lowest elevation position occupied during the sampling period.

WSN	Sex	SVL	Mass	Days	Mean ND	SD ND	Max ND	Mean elevation	SD elevation	Peak date
**WSN 16**	F	43.5	4.7	71	398.78	410.87	1285.70	1503.03	118.01	2021-05-07
**WSN 18**	F	51.8	4.56	82	193.96	222.61	981.55	1579.84	73.27	2021-05-23
**WSN 23**	F	41.2	3.7	41	188.08	286.68	998.61	1553.36	102.95	2021-05-14
**WSN 28**	M	60.24	6.5	80	58.00	37.09	326.48	1602.07	9.99	-
**WSN 29**	F	43	4.57	49	399.52	191.77	1124.93	1556.44	125.10	2021-05-24
**WSN 31**	F	42.3	2.2	65	386.82	309.04	1160.69	1525.48	105.12	2021-05-04
**WSN 33**	F	44	4.32	49	112.44	211.71	1121.88	1595.56	59.57	2021-05-24
**WSN 39**	F	40	3.99	88	146.40	253.11	1215.78	1573.23	88.99	2021-05-22
**WSN 40**	F	44	4.87	82	446.90	524.07	1936.20	1594.11	73.00	2021-05-08
**WSN 46**	F	50.2	4.41	79	366.45	513.07	1385.97	1553.80	106.80	2021-05-11
**WSN 48**	M	53	5.16	86	50.99	75.44	567.01	1598.24	23.64	-
**WSN 53**	F	46.2	4.37	79	356.69	296.10	848.75	1526.84	136.86	2021-05-02
**WSN 54**	F	37.6	3.47	51	95.79	76.31	508.32	1589.22	27.65	2021-04-22
